# Prediction of short-term antidepressant response using probabilistic graphical models with replication across multiple drugs and treatment settings

**DOI:** 10.1038/s41386-020-00943-x

**Published:** 2021-01-15

**Authors:** Arjun P. Athreya, Tanja Brückl, Elisabeth B. Binder, A. John Rush, Joanna Biernacka, Mark A. Frye, Drew Neavin, Michelle Skime, Ditlev Monrad, Ravishankar K. Iyer, Taryn Mayes, Madhukar Trivedi, Rickey E. Carter, Liewei Wang, Richard M. Weinshilboum, Paul E. Croarkin, William V. Bobo

**Affiliations:** 1 Department of Molecular Pharmacology and Experimental Therapeutics, Mayo Clinic, Rochester, MN USA; 2 Department of Translational Research Psychiatry, Max Planck Institute of Psychiatry, Munich, Germany; 3 Duke-National University of Singapore, Singapore, Singapore; 4 Department of Psychiatry and Behavioral Sciences, Duke University School of Medicine, Durham, NC USA; 5 Department of Psychiatry, Texas Tech University-Health Sciences Center, Midland, TX USA; 6 Department of Health Sciences Research, Mayo Clinic, Rochester, MN USA; 7 Department of Psychiatry and Psychology, Mayo Clinic, Rochester, MN USA; 8 Garvan Institute of Medical Research, Sydney, NSW Australia; 9 Department of Statistics, University of Illinois at Urbana-Champaign, Champaign, IL USA; 10 Department of Electrical and Computer Engineering, University of Illinois at Urbana-Champaign, Champaign, IL USA; 11 Department of Psychiatry, University of Texas Southwestern Medical Center, Dallas, TX USA; 12 Department of Health Sciences Research, Mayo Clinic, Jacksonville, FL USA; 13 Department of Psychiatry and Psychology, Mayo Clinic, Jacksonville, FL USA

**Keywords:** Translational research, Depression

## Abstract

Heterogeneity in the clinical presentation of major depressive disorder and response to antidepressants limits clinicians’ ability to accurately predict a specific patient’s eventual response to therapy. Validated depressive symptom profiles may be an important tool for identifying poor outcomes early in the course of treatment. To derive these symptom profiles, we first examined data from 947 depressed subjects treated with selective serotonin reuptake inhibitors (SSRIs) to delineate the heterogeneity of antidepressant response using probabilistic graphical models (PGMs). We then used unsupervised machine learning to identify specific depressive symptoms and thresholds of improvement that were predictive of antidepressant response by 4 weeks for a patient to achieve remission, response, or nonresponse by 8 weeks. Four depressive symptoms (depressed mood, guilt feelings and delusion, work and activities and psychic anxiety) and specific thresholds of change in each at 4 weeks predicted eventual outcome at 8 weeks to SSRI therapy with an average accuracy of 77% (*p* = 5.5E-08). The same four symptoms and prognostic thresholds derived from patients treated with SSRIs correctly predicted outcomes in 72% (*p* = 1.25E-05) of 1996 patients treated with other antidepressants in both inpatient and outpatient settings in independent publicly-available datasets. These predictive accuracies were higher than the accuracy of 53% for predicting SSRI response achieved using approaches that (i) incorporated only baseline clinical and sociodemographic factors, or (ii) used 4-week nonresponse status to predict likely outcomes at 8 weeks. The present findings suggest that PGMs providing interpretable predictions have the potential to enhance clinical treatment of depression and reduce the time burden associated with trials of ineffective antidepressants. Prospective trials examining this approach are forthcoming.

## Introduction

**Fig. 1:**
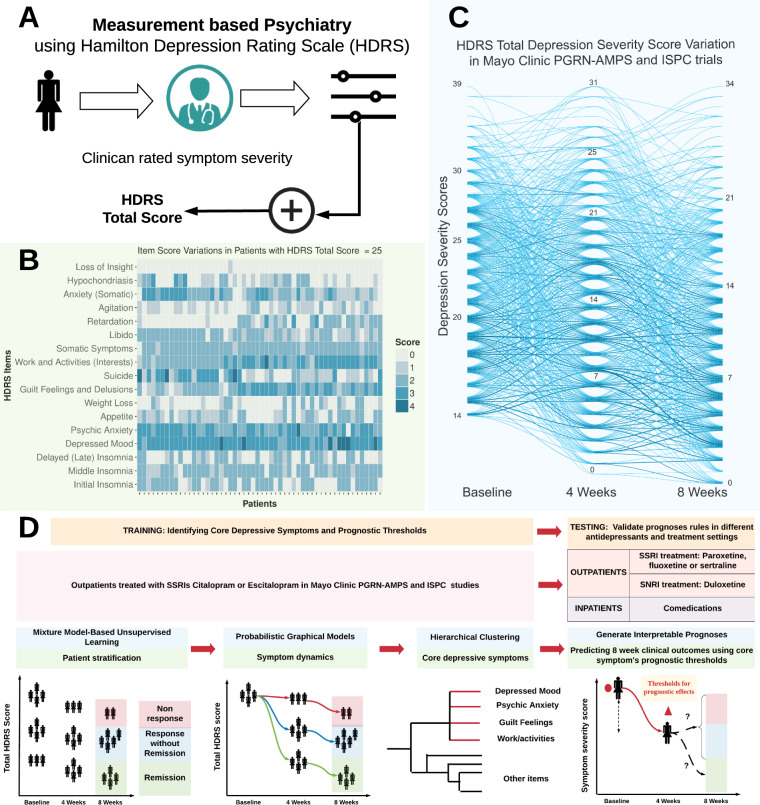
Study overview. **A** Measurement-based psychiatry using validated rating scales such as the 17-item Hamilton Depression Rating Scale (HDRS) to measure severity of depression symptoms. HDRS total score is sum of severity of individual HDRS item (depressive symptom). **B** Heterogeneity of symptom severity in the training datasets (Mayo Clinic PGRN-AMPS and ISPC subjects) with HDRS total score of 25 at baseline. **C** Heterogeneity in longitudinal variations of HDRS total score in Mayo Clinic PGRN-AMPS and ISPC subjects treated with citalopram/escitalopram. **D** Proposed analyses workflow to build probabilistic graphical model (PGM) and derive individualized prognoses of treatment outcomes at 8 weeks using changes in severity of focused set of depressive symptoms between baseline and after 4 weeks of antidepressant treatment.

Major depressive disorder (MDD) is a complex disease comprising several symptoms related to mood, capacity to derive pleasure, physical status, and cognitive functioning [1]. Despite variable efficacy rates [2], antidepressants are the most-commonly used treatments for MDD. Therapeutic responses to antidepressants can be reliably measured using validated rating scales (See Fig. 1A), which can then be used as a guide for clinical decision making [3, 4]. However, there are no validated quantitative prognostic “symptom level” indicators that can be used to operationalize decisions about continuing or changing treatment based on the most-likely eventual treatment outcome. The high variability of depressive symptom presentations (See Fig. 1B) and clinical trajectories of MDD (See Fig. 1C) present formidable challenges for clinician decision making [5]. As a consequence, antidepressant treatment selection occurs on a “try-and-try-again” basis, based on lack of perceived treatment benefit by patients and clinicians [6]. Hence, there is a significant need to derive accurate and quantitatively-based prognoses of eventual treatment outcomes, given a set of measured changes in symptom severity at an intermediate timepoint [7, 8], before therapeutic trials are declared to be fully complete, usually after 8 weeks of treatment [9–12].

Prior studies using STAR*D and other large datasets have investigated whether early improvements in total depression rating scale scores can be used to predict eventual treatment nonresponse [13], which would enable a change in treatment. These studies relied on the use of growth mixture models and trajectory analyses [14–16] which do not provide easily interpretable prognoses (prediction) of eventual treatment outcomes using specific patterns of improvement in depressive symptoms at intermediate treatment timepoints. These prior studies showed that, as would be expected, early response (i.e., >50% reduction in total depression severity scores at 4 weeks) is prognostic of response at 8 weeks, and a <20% reduction in total depression severity at 4 weeks is prognostic of nonresponse at 8 weeks. However, this observation accounts for variations in less than half the patients across the studies, and in the remaining majority of the patients, there is still significant heterogeneity in the 8-week outcomes of patients who are nonresponders at 4 weeks. Hence, the need for conditioning the likelihood of 8-week treatment outcomes on early improvements in individual depressive symptoms in conjunction with changes in total depression severity is highlighted by the observation that nearly half of nonresponders to antidepressant therapy (i.e., <50% reduction in total depression severity scores) at 4 weeks are eventual responders to therapy at 8 weeks [17].

Antidepressant response is probabilistic in nature (i.e., longitudinal variations in MDD severity and treatment outcomes vary in patients who begin treatment with the same MDD severity). Hence, we examined whether mathematical formulations such as probabilistic graphical models (PGMs) [18] that allow for reasoning under conditions of uncertainty, could thus be suitable methods to derive interpretable prognoses of antidepressant response. Specifically, we used PGMs in conjunction with unsupervised machine learning methods to derive interpretable and accurate prognoses of antidepressant treatment outcomes first in a training dataset (see Fig. 1D), then through replication using other datasets. We hypothesized that a PGM-based model would result in significantly higher accuracy for the short-term prediction of response to antidepressants in adults with MDD, and achieve replications across multiple classes of antidepressants and treatment settings, compared with approaches that incorporated only baseline clinical and sociodemographic predictor variables.

## Materials and methods

### Data sources

The datasets used for this study (described below and in Supplementary Methods) included subjects that met DSM-IV criteria for nonpsychotic MDD, confirmed using modules A, B (screen-only version), and D of the Structured Clinical Interview for DSM-IV (SCID). Subjects received at least 8 weeks of treatment with a study drug (see Supplementary Table 1), i.e., selective serotonin reuptake inhibitors (SSRIs), serotonin-norepinephrine reuptake inhibitors (SNRIs) or tricyclic antidepressants (TCAs). Depressive symptoms were measured using the 17-item clinician rated version of Hamilton Depression Rating Scale (HDRS) at baseline, 4 weeks, and 8 weeks. Participation in each of the studies required IRB approval at their respective institutions.

#### Training datasets

We used data from 947 MDD patients treated with SSRIs (citalopram/escitalopram) in two large, nonoverlapping clinical trial datasets from the Mayo Clinic Pharmacogenomics Research Network (PGRN-AMPS [19]) and the International SSRI Pharmacogenomics Consortium (ISPC [20]) to develop the PGM and derive prognoses rules.

#### Testing datasets

We then tested the prognostic capabilities of our model using datasets from independent cohorts of MDD patients as described:

- Paroxetine, fluoxetine, sertraline (248 ISPC outpatients), or escitalopram (216 outpatients from a pooled dataset obtained from Eli Lilly and Co.);
- Duloxetine (1067 outpatients from pooled datasets from Eli Lilly and Co.); and
- Combination pharmacotherapy with an SSRI or SNRI plus a TCA (465 hospitalized participants in the Munich Antidepressant Response Signature [MARS [21]] Study).

#### Placebo data

Data from 575 patients who received a pill placebo was used for ascertaining the prognostic effects of depression symptoms that were most likely due to drug effects.

### Outcomes

The categorical treatment outcomes based on HDRS total scores were remission at 8 weeks (HDRS total score ≤7), response without remission (referred to as response; a >50% reduction in HDRS total score from baseline and HDRS total score >7), and nonresponse (<50% reduction in HDRS total score from baseline).

### Probabilistic graph: motivation and construction

The PGM in this study was composed of states (nodes representing MDD severity) at each treatment timepoint and probabilistic transitions between states (i.e., fraction of patients moving between states of one timepoint to states of the next timepoint). To demonstrate the complexity of comprehending antidepressant response from a clinician’s perspective, let the states of the PGM be *N* unique total HDRS scores observed at each treatment timepoint *t*. Then, for each treatment timepoint (*t*), the number of trajectories of scores is proportional to *N*^*t*^. As shown in Fig. 1C, such a complex array of trajectories is difficult to interpret and is of little clinical value for estimating treatment outcomes.

To derive a more compact representation of antidepressant response trajectories, *t* could not be reduced because the follow-up timepoints were fixed; thus, we endeavored to reduce *N* by stratifying patients. With the exception of remission at 8 weeks, there was no natural definition of patient stratification at other timepoints as defined by the range of HDRS scores. We used unsupervised learning (specifically, Gaussian mixture models) to infer patient subgroups, as described in our prior work [22]. Gaussian mixture models were chosen because of inherent latent structures in the distribution of depression severity scores (i.e., the distribution of scores was likely characterized by multiple Gaussian curves). Inputs to the Gaussian mixture models were HDRS total scores from each timepoint from PGRN-AMPS and ISPC subjects treated with citalopram or escitalopram. Using Bayesian information criteria to test goodness of fit, the Gaussian mixture models algorithmically identified the minimum number of Gaussians (i.e., strata) that best approximated the actual distribution of total depression severity scores. Patients were assigned to strata that maximized the evaluation of the learned Gaussian function parameters (i.e., mean and standard deviation). Using this algorithmic formulation, three strata of patients (patient clusters) were inferred in the training datasets based on total HDRS scores at baseline, 4 weeks and 8 weeks [22]. The strata (described in Supplementary Table 2), are named by a letter-number tuple. The letters (e.g., A, B, and C) represent the treatment timepoints (baseline, 4 and 8 weeks, respectively), and the numeric suffix at each timepoint represents the level of depression severity, with “3” being the most severely depressed subjects and “1” being the least-severely depressed. The ranges of total HDRS scores for each cluster are shown below:

- Baseline stratifications: A1 [14–18], A2 [19–24], A3 [25–39];
- Week 4 stratifications: B1 [0–8], B2 [9–15], B3 [16–31]; and
- Week 8 stratifications: C1 [0–7], C2 [8–15], and C3 [16–34].

The strata inferred at 8 weeks (C1, C2, and C3) had acceptable clinical validity, given that all patients in the C1 stratum achieved remission and all patients in the C3 stratum were nonresponders. Eighty-seven percent of patients in the C2 stratum achieved response without remission and the remaining 13% were nonresponders.

In the absence of clustering, there were 680 unique MDD response trajectories among the 947 subjects in the training datasets (see Fig. 1C). With the use of patient clustering and stratification at each treatment timepoint, the number of MDD response trajectories reduced to a maximum of 27 paths (i.e., *N* = 3, and *t* = 3, and *N*^*t*^ = 3^3^ = 27). We then modeled the most-likely variations in depression severity along these paths for patients, starting from a given baseline stratum.

### Probabilistic graph and path probabilities

A hidden Markov model (HMM) with forward transitions was formulated to derive the trajectories of change in MDD severity in the training datasets. For the treatment timepoints (baseline, 4 and 8 weeks), the HMM was characterized by (1) hidden states (patient strata defined by range of total HDRS score, inferred from the study data); (2) observation states at 4 and 8 weeks (categorical response defined by HDRS total scores, based on transitions between hidden states of one timepoint to the next); and (3) forward transition probabilities (fraction of patients moving between strata of one timepoint to the next timepoint). The forward algorithm was used to derive the likelihood for all paths that originated from a given stratum at baseline, and terminated in a stratum at 8 weeks. By using the forward algorithm, we did not have to condition the trajectories originating from a baseline stratum based on an outcome of interest at 8 weeks. For every pair of strata at baseline and 8 weeks, the paths that had the highest likelihood and at least 10% of the patients from the baseline strata (tabulated in Supplementary Table 2) were chosen as the symptom dynamic paths.

### Prognostic symptoms and prognoses rules

**Fig. 2:**
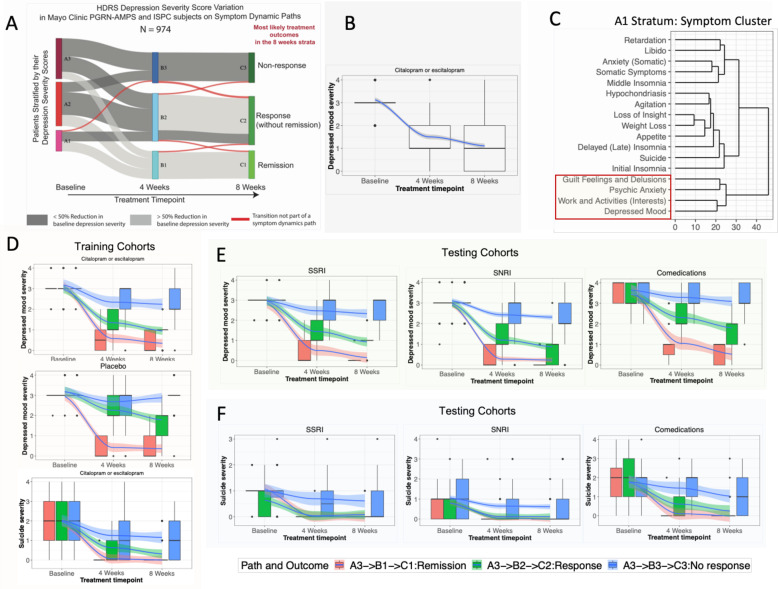
Schematic of symptom dynamic paths and prognostic depressive symptoms. **A** Symptom dynamic paths in patients in the training datasets (Mayo PGRN-AMPS and ISPC subjects). **B** Longitudinal variation in severity score of depressed mood (HDRS item) in patients starting in the A3 stratum at baseline. **C** Symptom clusters within patient strata (e.g., A1 at baseline), illustrating the grouping of prognostic symptoms. Fig. **B**, **D**, **E**, and **F** depict variations in depressed mood (prognostic symptom) and suicide ideation (nonprognostic symptom) in patients with antidepressants or placebo on symptom dynamic paths A3 → B3 → C3 (nonresponders at 8 weeks), A3 → B2 → C2 (responders without remission at 8 weeks), and A3 → B1 → C1 (remission at 8 weeks). In Fig. **B**, **D**, **E** and **F**, the solid blue lines in each figure represent the variations (mean changes) in prognostic symptom scores, and shaded regions around the mean illustrate their 95% confidence intervals (CIs). The boxplots and error bars represent the overall variability in prognostic symptom severity scores at each timepoint. Fig. **B** and **D**: Comprise all patients originating in stratum A3 in training and placebo datasets. The variations in prognostic and nonprognostic symptoms in testing data cohorts are visualized in Fig. **E** and **F**, respectively.

We sought to identify a group of *prognostic symptoms* that had (a) non-zero symptom severities at baseline across the majority of patients (to assess the quantum of early reductions in severity during treatment for predicting long-term response; see Supplementary Methods for details), (b) similar symptom severity scores (creating symptom clusters derived using hierarchical clustering for each stratum; illustrated in Fig. 2C (symptom clusters for A1 stratum) and Supplementary Fig. 1 (symptom clusters for all strata) at all timepoints on symptom dynamic paths originating from a baseline stratum (to establish how many symptoms with similar severity at baseline should improve at 4 weeks for predicting 8-week outcomes), and (c) different distributions of symptom severity scores between symptom dynamic paths (to quantify the level of change in a group of symptoms at 4 weeks needed to achieve specific outcomes at 8 weeks). These criteria allowed us to identify a group of depressive symptoms that had similar severities at baseline (criteria (a) and (b)) and across all treatment timepoints (a grouping effect) and had different levels of severity between individual symptoms dynamic pathways (a discriminatory effect, with criterion (c)).

The thresholds of change in prognostic symptom severity were derived based on absolute difference in median scores on symptom dynamic paths between baseline and 4-week strata (see Supplementary Table 3). Chi-square tests were used to identify the minimum number of prognostic symptoms needed to exceed (or not exceed) thresholds at 4 weeks to be prognostic of outcomes at 8 weeks (see Supplementary Methods for details). We then computed the accuracy (i.e., fraction of patients for whom the prognoses rule predicted the correct treatment outcome) and odds ratio (OR) of the most-likely outcome expected at 8 weeks in patients who transitioned from a baseline stratum to a stratum at 4 weeks (also tabulated in Table 1). The OR represents the odds that the expected treatment outcome at 8 weeks will occur if patients are covered by the prognoses rule, compared to the odds of the same outcome occurring in patients not covered by the prognoses rule. The statistical significance (*p* value) of the prognostic accuracies derived using prognostic symptoms was established by comparing the observed accuracy against the null information rate (NIR)—a proxy for chance. The NIR of 0.53 represents the fraction of subjects in the training datasets for whom (i) baseline clinical and sociodemographic factors as predictors accurately predicted their treatment outcomes using Random Forests (derived from our prior work [23]), and (ii) categorical non-responder status at 4 weeks correctly predicted 8-week outcomes (i.e., only 53% of the 514 subjects in our training data who were nonresponders at 4 weeks [<50% improvement in total HDRS score from baseline] were responders at 8 weeks). Finally, we used Kolmogorov–Smirnov (for age) and Chi-square tests (for sex and race) to evaluate if prognosis rules or accuracies were associated with age, sex, or race (the common sociodemographic factors across all datasets).

**Table 1:** Accuracy of prognoses rules.

Training (PGRN-AMPS and ISPC): SSRI (Citalopram/Escitalopram) *N* = 947 outpatients										
Baseline strata	4-week strata	Number of patients making transition	Most-likely outcome	Prognoses Rule and Coverage			Probability of most-likely outcome (accuracy = 100*probabilty)	Odds ratio (OR)	95% confidence interval	*p* value of accuracy with NIR = 0.53
				Change in symptom severity (Baseline—4 week)	Number of symptoms needing the change	Coverage (Fraction of patients covered by prognoses rule)				
A3	B3	96	Nonresponse	≤1	≥3	0.75	0.60	6.90	(2.03, 23.74)	0.05
	B2	104	Response	≥2	≥2	0.65	0.85	3.27	(1.26, 8.5)	4.83E-13
	B1	59	Remission	≥2	≥2	0.86	0.75	3.63	(0.9, 16)	5.64E-07
A2	B3	88	Nonresponse	≤1	≥3	0.87	0.80	7.70	(1.46, 40.41)	1.12E-09
	B2	148	Response	≥1	≥2	0.95	0.85	5.40	(2.3, 12.87)	4.83E-13
	B1	102	Remission	≥2	≥2	0.88	0.75	2.20	(0.6, 7.66)	5.64E-07
A1	B2 or B3	100	Nonresponse	≤1	≥3	0.94	0.70	4.71	(0.81, 27.23)	7.85E-05
	B1	160	Remission	≥2	≥1	0.93	0.82	4.35	(1.18, 16)	6.15E-11
**Testing (ISPC+Eli Lilly): SSRIs (Escitalopram, fluoxetine, sertraline, paroxetine)** ***N*** = 464 outpatients										
A3	B3	82	Nonresponse	≤1	≥3	0.89	0.72	5.30	(1.2, 23.2)	1.26E-05
	B2	63	Response	≥2	≥2	0.86	0.86	6.50	(1.3, 33)	8.28E-14
	B1	41	Remission	≥2	≥2	0.83	0.70	6.00	(1, 36.3)	7.85E-05
A2	B3	39	Nonresponse	≤1	≥3	0.87	0.85	12.60	(1.14, 65.9)	4.83E-13
	B2	98	Response	≥1	≥2	0.80	0.62	4.10	(1.13, 12.7)	0.02
	B1	60	Remission	≥2	≥2	0.67	0.63	2.50	(0.84, 7.6)	0.01
A1	B2 or B3	33	Nonresponse	≤1	≥3	0.85	0.70	7.20	(0.64, 82)	7.85E-05
	B1	48	Remission	≥2	≥1	0.67	0.84	4.20	(1.1, 16.5)	2.61E-12
**Testing (Eli Lilly): SNRI (Duloxetine)** ***N*** = 1067 outpatients										
A3	B3	201	Nonresponse	≤1	≥3	0.86	0.73	1.80	(1, 4.2)	4.69E-06
	B2	125	Response	≥2	≥2	0.65	0.84	2.80	(1.2, 6.8)	2.61E-12
	B1	82	Remission	≥2	≥2	0.56	0.83	2.30	(0.9, 6.5)	1.31E-11
A2	B3	156	Nonresponse	≤1	≥3	0.95	0.78	9.10	(1.6, 9.14)	1.59E-08
	B2	237	Response	≥1	≥2	0.95	0.62	2.20	(1.2, 4.04)	0.02
	B1	129	Remission	≥2	≥2	0.67	0.77	2.14	(0.93, 4.9)	5.51E-08
A1	B2 or B3	89	Nonresponse	≤1	≥3	0.77	0.63	5.05	(1.4, 17.9)	0.01
	B1	48	Remission	≥2	≥1	0.88	0.83	25.00	(1.9, 35)	1.31E-11
**Testing (MARS): COMEDICATIONS** ***N*** = 465 inpatients										
A3	B3	57	Nonresponse	≤1	≥3	0.88	0.66	4.80	(0.9, 27)	0.002
	B2	71	Response	≥2	≥2	0.76	0.73	2.80	(0.84, 8.3)	4.69E-06
	B1	29	Remission	≥2	≥2	0.84	0.69	9.00	(0.9, 91)	0.0002
A2	B3	51	Nonresponse	≤1	≥3	0.86	0.76	4.40	(1, 23)	1.81E-07
	B2	101	Response	≥1	≥2	0.80	0.60	3.25	(1, 10.6)	0.06
	B1	50	Remission	≥2	≥2	0.56	0.57	2.90	(1.1, 8.9)	0.1933479
A1	B2 or B3	49	Nonresponse	≤1	≥3	0.88	0.76	12.80	(0.74, 37)	1.81E-07
	B1	57	Remission	≥2	≥1	0.75	0.57	4.60	(1.1, 19)	0.2
**Prognoses performance in placebo-treated patients (Eli Lilly** ***N*** = 575)										
A3	B3	96	Nonresponse	≤1	≥3	0.95	0.80	0.40	(0.03, 4.9)	0.60
	B2	104	Response	≥2	≥2	0.40	0.69	2.00	(0.45, 8.9)	0.85
	B1	59	Remission	≥2	≥2	0.95	0.73	1.40	(0.1, 19)	0.75
A2	B3	88	Nonresponse	≤1	≥3	0.96	0.87	0.20	(0.02, 3.4)	0.80
	B2	148	Response	≥1	≥2	0.87	0.47	0.48	(0.13, 1.8)	0.85
	B1	102	Remission	≥2	≥2	0.86	0.67	1.20	(0.26, 5.7)	0.75
A1	B2 or B3	100	Nonresponse	≤1	≥3	0.86	0.67	0.67	(0.23, 1.92)	0.70
	B1	160	Remission	≥2	≥1	0.91	0.71	2.50	(0.7, 13.85)	0.82

Prognoses performance of prognostic symptoms in patients making specific transitions between baseline and 4-week strata. The ranges of depression severity scores in each strata are as follows: A1 [14–18], A2 [19–24], A3 [25–39]; B1 [0–8], B2 [9–15], B3 [16–31]. The OR represents the odds that the expected treatment outcome at 8 weeks will occur if patients are covered by the prognoses rule, compared to the odds of the same outcome occurring in patients not covered by the prognoses rule. The statistical significance (*p*-value) of the prognoses’ accuracy was established using the null information rate (NIR).

## Results

### Symptom dynamic paths

For the patients treated with citalopram/escitalopram in the training dataset, specific symptom dynamic paths (Fig. 2A) were derived (see Supplementary Table 2 for likelihood scores for symptom dynamic paths). Patients starting in any stratum at baseline were most likely to achieve remission at 8 weeks if they transitioned into the B1 stratum at 4 weeks, and the clinical observation at 4 weeks was response. Patients starting in the A2 or A3 strata at baseline were most likely to achieve response at 8 weeks if they transitioned into the B2 stratum at 4 weeks and the clinical observation at 4 weeks was response; and were most likely to be nonresponders at 8 weeks if they transitioned into the B3 stratum at 4 weeks and the clinical observation at 4 weeks was also a nonresponse. Patients starting in the A1 stratum at baseline were most likely to be nonresponders at 8 weeks if they transitioned into the B2 stratum at 4 weeks and the clinical observation at 4 weeks was also nonresponse. There was no symptom dynamic path between A1 to C3 since fewer than 8% of the patients reached the C3 stratum at 8 weeks via either the B3 or the B2 strata at 4 weeks.

### Prognostic symptoms

Four HDRS items (depressed mood, psychic anxiety, guilt feelings/delusions, and work/activities) met the prognostic symptom criteria for patients in the training dataset. We illustrate the variations in severity of prognostic symptoms in patients with and without the superimposition of symptom dynamic paths (e.g., for depressed mood, see Fig. 2B, D), using data from subjects originating in A3 stratum at baseline. Improvement in the severity of depressed mood can be visualized at 4 and 8 weeks in Fig. 2B, but there is still a high degree of interpatient variation in the scores for depressed mood (as shown by the large spread of boxplots) when subjects are not stratified and analyzed using symptom dynamic paths. By stratifying patients and deriving symptom dynamic paths (e.g., those originating from stratum A3, as shown in Fig. 2D), the discriminatory effect of scores at 8 weeks was better reflected in the patterns of response at 4 weeks. No such discriminatory effects occur for nonprognostic symptoms, as shown in Supplementary Fig. 2. No prognostic symptoms could be identified for patients who received placebo using only the prognostic symptom criteria (see Fig. 2D).

### Prognostic performance of prognostic symptoms in training dataset

**Fig. 3:**
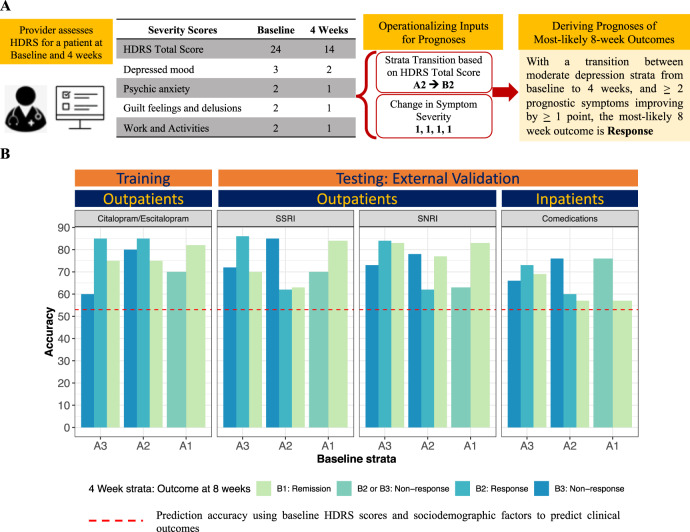
Prognosis rules and their predictive accuracies. **A** Demonstration of the operationalization of prognoses rules to predict 8-week treatment outcome. **B** For each of the baseline and 4-week strata, we illustrate the accuracy of the prognoses in comparison with the average prediction accuracy (53% in dashed red line) that is achieved when using only baseline clinical and sociodemographic factors as predictors.

We illustrate the operationalization of deriving prognoses using changes in total HDRS and prognostic symptoms in Fig. 3A. The prognostic performance of the changes in prognostic symptoms at 4 weeks for predicting 8-week outcomes in citalopram- or escitalopram-treatment patients are summarized below, and are shown in Fig. 3B and Table 1:

- For patients originating in the A3 stratum: (1) the accuracy in the prediction of nonresponse at 8 weeks was 60% (OR 6.9, CI 2.03–23.74, *p* = 0.05) by transitioning into the B3 stratum with ≥3 prognostic symptoms improved by ≤1 point at 4 weeks; (2) the accuracy in the prediction of response at 8 weeks was 85% (OR 3.27, CI 1.26–8.5, *p* = 4.83E-13) by transitioning into the B2 stratum with ≥2 prognostic symptoms improved by ≥2 points at 4 weeks; and (3) the accuracy in the prediction of remission at 8 weeks was 70% (OR 2.4, CI 0.8–19, *p* = 5.64E-7) by transitioning into the B1 stratum with ≥2 prognostic symptoms improved by ≥2 points at 4 weeks.
- For patients originating in the A2 stratum: (1) the accuracy in the prediction of nonresponse at 8 weeks was 80% (OR 7.7, CI 1.46–40.1, *p* = 1.12E-9) by transitioning into the B3 stratum with ≥3 prognostic symptoms improved by ≤1 point at 4 weeks; (2) the accuracy in the prediction of response at 8 weeks was 85% (OR 5.4, CI 2.3–12.87, *p* = 4.83E-13) by transitioning into the B2 stratum with ≥2 prognostic symptoms improved by ≥1 point at 4 weeks, and (3) the accuracy in the prediction of remission at 8 weeks was 75% (OR 2.2, CI 0.6–7.66, *p* = 5.64E-7) by transitioning into the B1 stratum with ≥2 prognostic symptoms improved by ≥2 points at 4 weeks.
- For patients originating in the A1 stratum: (1) the accuracy in the prediction of nonresponse at 8 weeks was 70% (OR 4.71, CI 0.81–27.23, *p* = 7.85–5) by transitioning into the B2 or B3 stratum with ≥3 prognostic symptoms improved by ≤1 point at 4 weeks; and (2) the accuracy in the prediction of remission at 8 weeks was 82% (OR 4.35, CI 1.18–16, *p* = 6.15E-11) by transitioning into the B1 stratum with ≥1 prognostic symptoms improved by ≥2 points at 4 weeks.

The criteria for minimum number of prognostic symptoms needed for threshold rules to be met was applicable in over 67% (see coverage column in Table 1) of the patients starting from any of the baseline strata. There were no associations with age, sex, or race for meeting the prognostic symptom criteria or accuracy of prognoses. The observed outcome was nonresponse for nearly all (92%) of the remaining patients.

### Replication of prognostic performance of prognostic symptoms in testing datasets

We first assigned patients in the testing datasets who were treated with SSRIs, duloxetine, and combination therapy to a stratum at each timepoint, as defined by the same range of total HDRS scores derived from the training dataset. As shown in Fig. 2E, F, prognostic and nonprognostic symptom variations in the testing datasets (see Fig. 2E, F) were similar to those of the training dataset (see Fig. 2D). We then calculated the accuracies of forecasted outcomes at 8 weeks (see Fig. 3B) using the same prognostic thresholds of prognostic symptom changes at 4 weeks derived from the training cohort (additional details in Table 1). Prognoses performance of the change in prognostic symptoms at 4 weeks for predicting 8-week outcomes in the testing datasets are summarized below, and are shown in Table 1:

- For patients originating in the A3 stratum: (1) the accuracies in the prediction of nonresponse at 8 weeks were 66%, 73%, and 67%, respectively, for patients treated with other SSRIs, duloxetine, and combination therapy who transitioned to the B3 stratum with ≥3 prognostic symptoms improved by ≤1 point at 4 weeks; (2) the accuracies in the prediction of response at 8 weeks were 88%, 84%, and 73%, respectively, for patients who transitioned to the B2 stratum with ≥2 prognostic symptoms improved by ≥2 points at 4 weeks; and (3) the accuracies in the prediction of remission at 8 weeks were noncalculable (due to lack of samples), 83% and 69% (*p* ≤ 0.08), respectively, for patients who transitioned to the B1 stratum with ≥2 prognostic symptoms improved by ≥2 points at 4 weeks.
- For patients originating in the A2 stratum: (1) the accuracies in prediction of nonresponse at 8 weeks was 93%, 78%, and 76%, respectively, for patients treated with other SSRIs, duloxetine, and combination therapy who transitioned to the B3 stratum with ≥3 prognostic symptoms improved by ≤1 point at 4 weeks; (2) the accuracies in the prediction of response at 8 weeks was 63%, 62%, and 68%, respectively, for patients who transitioned to the B2 stratum with ≥2 prognostic symptoms improved by ≥1 point at 4 weeks; and (3) the accuracies in prognoses of remission at 8 weeks was 72%, 77%, and 57%, respectively, for patients who transitioned to the B1 stratum with ≥2 prognostic symptoms improved by ≥2 points at 4 weeks.
- For patients originating in the A1 stratum*:* (1) the accuracies in the prediction of nonresponse at 8 weeks was 72%, 63%, and 76%, respectively, for patients treated with other SSRIs, duloxetine, and combination therapy who transitioned to the B2 or B3 stratum with ≥3 prognostic symptoms improved by ≤1 point at 4 weeks; (2) the accuracies in the prediction of remission at 8 weeks was 86%, 83%, and 57%, respectively, for patients who transitioned to the B1 stratum with ≥1 prognostic symptom improved by ≥2 points at 4 weeks.

Analogous to the case in the training dataset, the minimum prognostic symptom criteria captured variations in ≥71% of patients from each baseline cluster across all of the testing datasets, and sex was not associated with chances of meeting the prognostic symptom criteria or the prognoses accuracy. Nearly all (95%) of the remaining of patients had nonresponse as their outcome.

### Lack of prognostic symptoms and prognoses in placebo-treated patients

Prognostic depressive symptoms could not be identified using the criteria specified earlier in patients who received placebo. Instead, in Table 1, we report the accuracy and odds of outcomes in placebo patients (assigned to baseline and 4-week strata) using the four core HDRS-derived symptoms. The predictive accuracies in nearly all outcomes and the odds ratios for all outcomes were lower than those observed in escitalopram/citalopram-treated subjects from the training datasets (see Table 1). The only exception was that the odds ratio for predicting nonresponse was higher in placebo patients than escitalopram/citalopram-treated subjects.

## Discussion

We used probabilistic graphical models (PGMs) in conjunction with unsupervised machine learning methods to identify individual depressive symptoms that were highly predictive of antidepressant response, and thresholds of improvement needed in those symptoms by 4 weeks (an interim timepoint supported by treatment guidelines for making changes in antidepressant treatment [24–26]) to predict remission, response, or nonresponse by 8 weeks (which conservatively defines the end of a therapeutic antidepressant trial). The high levels of predictive accuracy achieved using a training dataset comprised of citalopram- or escitalopram-treated depressed outpatients replicated in three validation datasets that included depressed inpatients as well as outpatients treated with other SSRIs, duloxetine, and antidepressant combinations.

The prognostic depressive symptoms in this work were defined based on observed homogeneity in their responses at all timepoints, while demonstrating differential patterns of change under antidepressant treatment that were prognostic of clinical outcomes at 8 weeks. Whether they are core to the syndrome of MDD is a question not addressed in this work. However, there is a significant overlap of prognostic symptoms inferred in this work with symptoms in existing subscales (Maier-6 [27], Bech-6 [28], HAMD7 [29], and VQIDS-C_5_ [30]) that were derived from the full-scale HDRS or other rating scales to measure depressive symptoms that are more responsive to antidepressants and less-sensitive to their adverse effects. For example, the four prognostic symptoms derived from HDRS in this work were all included in Maier-6, Bech-6, and HAMD7. The prognostic depressive symptoms identified with QIDS-C in our study align with the items that were included in a brief version of QIDS-C [30] and with the “core emotional” symptoms of depression identified by others as being more responsive to citalopram/escitalopram treatment than were other depressive symptom clusters [14]. Our approach extends this prior work by establishing the prognostic capabilities of these symptoms using an unbiased approach.

The mathematical constructs of PGMs represent an analytical novelty in this work that permitted us to reason with uncertainty and overcome the challenges in interpreting longitudinal variations of antidepressant response when using other approaches, such as latent variable analyses with growth mixture models [14–16, 31–36]. For example, we used probabilistic graphs in this work instead of growth mixture models, given that growth mixture models (1) do not find paths algorithmically by conditioning upon improvements in symptoms at intermediate timepoints, (2) offer very limited interpretability of dynamics of symptom changes, and (3) need sufficient domain expertise to define the number of latent classes and trajectories, and ensure appropriate model fit, and then interpret the results [37–41] (which might prove challenging in analyses that are exploratory in nature). PGMs also provide an extendable analytical framework to derive antidepressant response trajectories for longer observation periods beyond 8 weeks, with the additional ability to identify interpretable response trajectories when the study timeline is a continuum (e.g., extracting visit data from electronic health records) as opposed to discrete timepoints (by formulating the PGM as a Markov jump process [42]). Deep learning approaches have been explored for inferring patient subgroups based on homogeneity in disease trajectories in a data-driven manner [43]. In fact, deep learning approaches and probabilistic graphs both have the advantage of high utility for modeling outcomes without requiring a prespecified number of trajectories. The advantage of PGMs over traditional deep learning or growth mixture model approaches lies in the mathematical formulation of PGMs that allow for reasoning with uncertainty and permits to conditioning future disease variations based on trajectories up to an interim timepoint. In our work, the forward algorithm construct in our PGM parallels the logical scheme used by clinicians in the measurement-based care of depressed patients. That is, the severity of depressive symptoms at baseline and changes in these symptoms are used to drive treatment decisions at an interim timepoint, prior to completion of a therapeutic antidepressant trial.

Based on the clinically-driven design of our PGM (incorporating change in depressive symptoms at 4 weeks in stratified patients), our approach could begin to inform the development of clinical decision support tools to augment (but not replace) practitioner expertise, improve patient engagement, and enhance shared decision-making by providing highly-interpretable quantitative prognostic information as a supplement to clinical judgment and patient preferences. Importantly, the PGM-based approach described in this work allows for the integration of biological measures, which may then be used to not only improve the predictability of antidepressant outcomes, but may also serve as a future strategy for individualizing choices of therapy for people with depression [23, 44]. Further work is needed to test the predictive capabilities of this approach, with the integration of biological measures, in prospective trials (NCT04355650), and in environments where measurement-based care of depressed patients is routinely delivered.

The consistently high predictive accuracies across numerous commonly-prescribed antidepressants observed in this work have several important implications that fit well with observations from the STAR*D trial: even with rigorously conducted antidepressant treatment, only 53% of patients may be expected to remit after 6 months [45–47]. By altering treatment at 4 weeks, an interim timepoint supported by practice guidelines and clinical evidence [9, 24, 25], a total of 2–4 months could be saved across two therapeutic trials that are each likely to fail after 8–12 weeks, a period of time that is often required for many depressed patients to remit or improve substantially [9, 47]. Our approach, which relied on only a limited number of depressive symptoms in addition to total depression scores to predict treatment outcomes, may introduce needed efficiencies into busy practices in addition to optimizing predictive accuracies. This feature may be especially important in busy primary care practices, and may hasten referrals for specialty mental health consultation or treatment if the predicted outcomes of treatment are nonresponse. As a cautionary note, we do not suggest that the full versions of depression rating scales be replaced with shorter versions based on prognostic symptoms only, which would fail to consider all of the important elements of MDD severity for individual patients, including suicidal ideation. Rather, our results suggest that focusing on early changes in prognostic symptoms may increase the prognostic value of full-scale depression measures, which were designed to measure disease severity but not necessarily to predict outcomes.

It is of significant interest that prognostic symptoms could not be identified in patients who received placebo. This observation is important because placebo response rates in clinical trials of antidepressants in MDD patients are high, ranging from 35 to 40% [48]. Moreover, prior applications of machine learning to large antidepressant clinical trial datasets have not shown systematic differences in the patterns of change in individual depressive symptoms over time between placebo and active treatment, even in placebo responders [14]. Although not a direct test of hypothesis (considering a relatively smaller number of placebo-treated subjects relative to those who received active treatment), our findings do suggest that the antidepressants we studied, as a group, exerted systematic effects on depressive symptoms that could not be demonstrated in placebo-treated subjects.

There are limitations to our study. Due to lack of data, we were unable to investigate whether changes in prognostic symptoms at timepoints earlier than 4 weeks can accurately predict clinical outcomes at 8 weeks, given evidence that eventual response may sometimes be predictable as early as 2 weeks [49]. The study data was restricted to three timepoints, which may not be sufficient to capture the full arc of the disease, including variations in depression severity and associated long-term outcomes that extend well beyond 8 weeks. There was no dose standardization across datasets, although this is less concerning given that drug dosage was not associated with clinical outcomes here or in previous studies [50]. Despite replication across independent testing datasets, additional studies are needed to establish the generalizability of our approach to other rating scales, medications and treatment approaches beyond those studied here, and longer follow-up durations. Our model, due to lack of data, does not account for the effects of nonadherence, comorbid diagnoses, environmental, and other socioeconomic factors. We were unable to address which treatments should be considered after failure to respond to a given medication due to the lack of sequential trial data. The impact of our findings on those who dropped out of treatment prior to 8 weeks is unknown because our analyses focused on trial completers. Finally, we did not have access to complete data on the number of previous therapeutic antidepressant trials for study patients, an important limitation given that the odds of achieving a positive treatment outcome with antidepressant treatment correlates inversely with the number of previous treatment failures [51].

In summary, this is the first study to examine PGMs in conjunction with unsupervised machine learning methods to derive interpretable and accurate prognoses of antidepressant treatment outcomes. The consistent results across several datasets from studies utilizing different antidepressant treatments and populations suggests this method to potentially utilize symptom trajectory improvements across time to provide much needed clinical decision support earlier in a patient’s treatment course.

## Funding and disclosure

MT: Consulting/advising ACADIA Pharmaceuticals, Alkermes Inc, Allergan, Alto Neuroscience Inc, Applied Clinical Intelligence LLC, Axsome Therapeutics, Boegringer Ingelheim, Engage Health Media, GreenLight VitalSign6 Inc, Janssen, Lundbeck Research USA, Navitor Pharmaceutical Inc, Otsuka, Perception Neuroscience, Pharmerit International, and SAGE Therapeutics. Edditorial Compensation from American Psychiatric Association (Deputy Editor for American Journal of Psychiatry), Oxford University Press. Receives funding from NIMH, NIDA, Patient-Centered Outcomes Research Institute (PCORI), Cancer Prevention Research Institute of Texas (CPRIT). AJR has received consulting fees from Akili, Brain Resource Inc., Compass Inc., Curbstone Consultant LLC, Emmes Corp., Johnson and Johnson (Janssen), Liva-Nova, Mind Linc, Otsuka America Pharmaceutical Inc., Sunovion; speaking fees from Liva-Nova; and royalties from Guilford Press and the University of Texas Southwestern Medical Center, Dallas, TX (for the Inventory of Depressive Symptoms and its derivatives). He is also named co-inventor on two patents: U.S. Patent No. 7,795,033: Methods to Predict the Outcome of Treatment with Antidepressant Medication, Inventors: McMahon FJ, Laje G, Manji H, Rush AJ, Paddock S, Wilson AS; and U.S. Patent No. 7,906,283: Methods to Identify Patients at Risk of Developing Adverse Events During Treatment with Antidepressant Medication, Inventors: McMahon FJ, Laje G, Manji H, Rush AJ, Paddock S. MAF has grant support from AssureRx, the Mayo Foundation, Myriad, the National Institute of Alcohol Abuse and Alcoholism (NIAAA), the National Institute of Mental Health (NIMH), and Pfizer, and consults for Janssen, Mitsubishi Tanabe Pharma Corporation, Myriad, Neuralstem Inc., Otsuka America Pharmaceutical, Sunovion, and Teva Pharmaceuticals. LW and RMW are co-founders and stockholders in OneOme LLC. WVB’s research has been supported by the National Institute of Mental Health, the Agency for Healthcare Quality and Research, and the Mayo Foundation for Medical Education and Research. He has contributed chapters to UpToDate concerning the use of antidepressants and atypical antipsychotic drugs for treating adults with bipolar major depression. APA receives support from the Mayo Foundation for Medical Education and Research. Rest of the authors have no conflicts to disclose. This material is based upon work partially supported by a Mayo Clinic and Illinois Alliance Fellowship for Technology-Based Healthcare Research; a CompGen Fellowship; an IBM Faculty Award; the National Science Foundation (NSF) under grants 2041339 and 1337732; the National Institutes of Health (NIH) under grants R01 AA27486, R01 MH113700, U19 GM61388, R01 GM28157, RC2 GM092729, R24 GM078233, RC2 GM092729, and T32 GM072474; and the Mayo Clinic Center for Individualized Medicine. Any opinions, findings, and conclusions or recommendations expressed in this material are those of the author(s) and do not necessarily reflect the views of the NSF or the NIH.

## Supplementary information

Supplementary Figure 1

Supplementary Figure 2

Supplementary File
