# Supplementary figures and images for: Prediction of short-term antidepressant response using probabilistic graphical models with replication across multiple drugs and treatment settings
Source: Neuropsychopharmacology. 2021 Jan 15;46(7):1272–82. doi: 10.1038/s41386-020-00943-x (PMC8134509; doi:10.1038/s41386-020-00943-x)

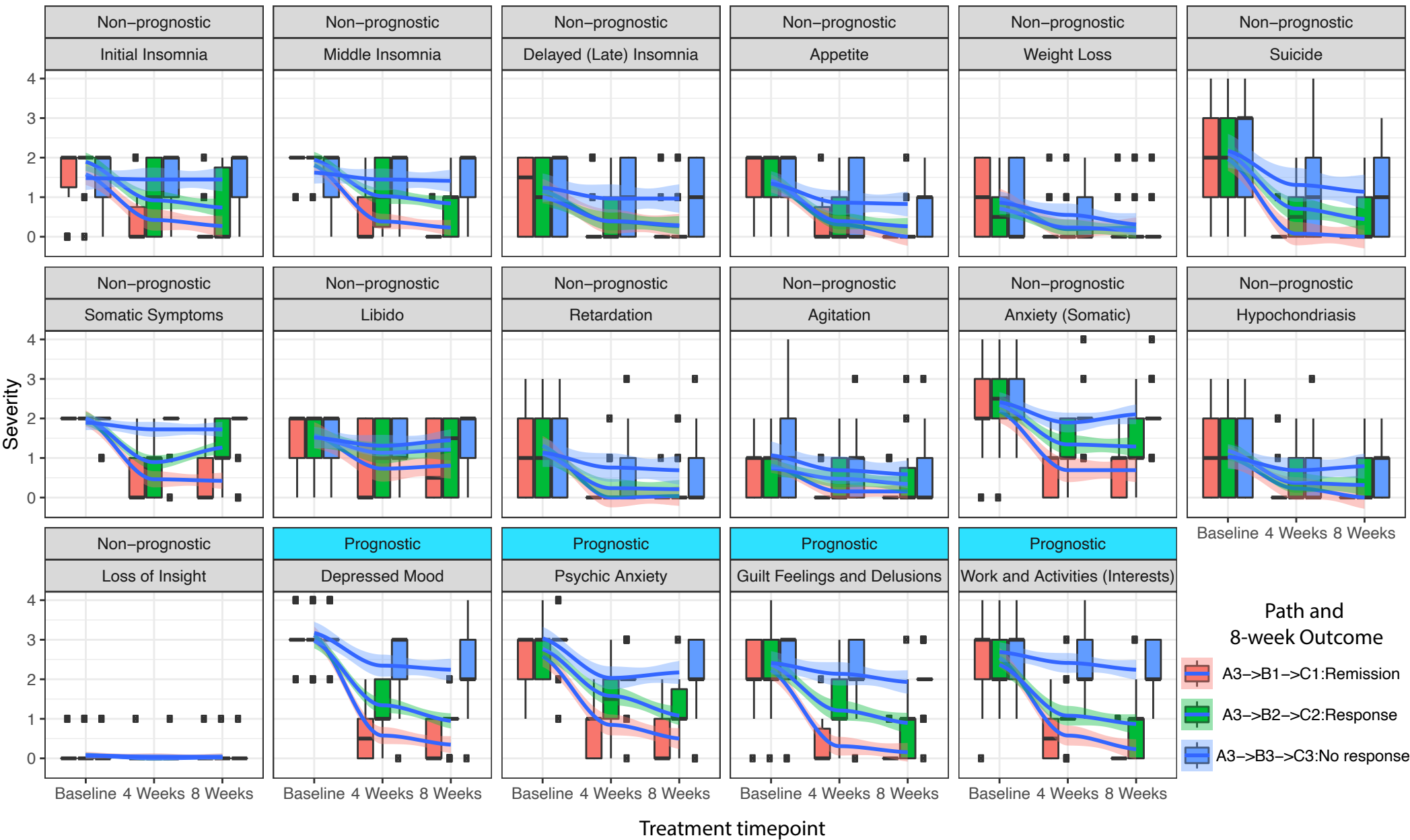

Supplement: Supplementary file 2 — Supplementary Figure 2 [file 41386_2020_943_MOESM2_ESM.pdf]
