# Supplementary material for: Prediction of short-term antidepressant response using probabilistic graphical models with replication across multiple drugs and treatment settings
Source: Neuropsychopharmacology. 2021 Jan 15;46(7):1272–82. doi: 10.1038/s41386-020-00943-x (PMC8134509; doi:10.1038/s41386-020-00943-x)
Supplement: Supplementary file 3 — Supplementary File [file 41386_2020_943_MOESM3_ESM.docx]

**SUPPLEMENTARY FILE**

**TITLE: Prediction of Short-Term Antidepressant Response Using Probabilistic Graphical Models with Replication Across Multiple Drugs and Treatment Settings**

**BYLINE: Probabilistic Graphs Predict Antidepressant Response**

**PREVIOUS PRESENTATION:** None

**AUTHORS:** Arjun P. Athreya MS, PhD^1^; Tanja Brückl, PhD^2^; Elisabeth B. Binder MD, PhD^2^; A. John Rush MD^3-5^; Joanna Biernacka PhD^6^; Mark A. Frye MD^7^; Drew Neavin BS, PhD^8^; Michelle Skime MS, CCRP^7^; Ditlev Monrad PhD^9^; Ravishankar K. Iyer PhD^10^; Taryn Mayes MS^11^, Madhukar Trivedi MD^11^; Rickey E. Carter PhD^12^; Liewei Wang MD, PhD^1^; Richard M. Weinshilboum MD^1^; Paul E. Croarkin, DO, MS^7^; and William V. Bobo MD, MPH^13^.

**AUTHOR AFFILIATIONS:**

^1^ Dept. of Molecular Pharmacology and Experimental Therapeutics, Mayo Clinic, Rochester, MN, USA.

^2^ Dept. of Translational Research Psychiatry, Max Planck Institute of Psychiatry, Munich, Germany.

^3^ Duke-National University of Singapore, Singapore.

^4^ Dept. of Psychiatry and Behavioral Sciences, Duke University School of Medicine, Durham, NC, USA.

^5^ Dept. of Psychiatry, Texas Tech University-Health Sciences Center, Midland, TX, USA.

^6^ Dept. of Health Sciences Research, Mayo Clinic, Rochester, MN, USA.

^7^ Dept. of Psychiatry and Psychology, Mayo Clinic, Rochester, MN, USA.

^8^ Garvan Institute of Medical Research, Sydney, New South Wales, Australia

^9^ Dept. of Statistics, Univ. of Illinois at Urbana-Champaign, IL, USA.

^10^ Dept. of Electrical and Computer Engineering, Univ. of Illinois at Urbana-Champaign, IL, USA.

^11^ Dept. of Psychiatry, Univ. of Texas Southwestern Medical Center, Dallas, TX, USA.

^12^ Dept. of Health Sciences Research, Mayo Clinic, Jacksonville, FL, USA.

^13^ Dept. of Psychiatry and Psychology, Mayo Clinic, Jacksonville, FL, USA.

**Corresponding Author:**

William V. Bobo, MD, MPH

Chair and Professor,

Dept. of Psychiatry and Psychology,

Mayo Clinic, Jacksonville, Florida.

[Bobo.william@mayo.edu](mailto:Bobo.william@mayo.edu)

PH: +1-904-953-7286

**FIGURE LEGENDS**

**Supplementary Fig. 1:** The hierarchical trees of the clusters of symptoms at baseline for patient strata in the training datasets (PGRN-AMPS and ISPC subjects) that were derived using HDRS symptom severity scores show consistent clustering of prognostic symptoms at all three time-points.

**Supplementary Fig. 2:** Longitudinal variation in severity score of prognostic and non-prognostic symptoms in patients starting in the A3 stratum at baseline and taking symptom dynamic paths A3 🡪 B3 🡪 C3 (non-responders at 8 weeks), A3 🡪 B2 🡪 C2 (responders without remission at 8 weeks), and A3 🡪 B1 🡪 C1 (remission at 8 weeks). The solid blue lines in each figure represent the variations (mean changes) in prognostic symptom scores, and shaded regions around the mean illustrate their 95% confidence intervals (CIs). The box plots and error bars represent the overall variability in prognostic symptom severity scores at each time-point.

**SUPPLEMENTARY TABLES**

**Supplementary Table 1:** Summary of patient data used in the study.

| Data Usage | Training | | Testing (External Validation) | | | | |
| --- | --- | --- | --- | --- | --- | --- | --- |
| Trial/Data Source | PGRN-AMPS | ISPC | ISPC | Eli Lilly | | MARS | Eli Lilly |
| Antidepressant Class | SSRI | | SSRI | | SNRI | SSRI + SNRI | Placebo |
| Treatment | Citalopram/  Escitalopram | | Fluoxetine, Paroxetine, Sertraline | Escitalopram | Duloxetine | Comedications |  |
| Sample size (N) | 603 | 344 | 248 | 216 | 1,067 | 465 | 575 |
| Age at enrollment, mean (SD) in years | 40 (13.52) | 44 (14.44) | 41.8 (14.2) | 46.5 (13.9) | 42 (13.45) | 46.5(14) | 51.9 (17.2) |
| Sex, n (%) |  |  |  |  |  |  |  |
| Women | 381 (63.2) | 237 (68.9) | 164 (66.1) | 151 (70) | 681 (63.8) | 250 (53.7) | 216 (37.6) |
| Men | 222 (36.8) | 107 (31.1) | 84 (33.9) | 65 (30) | 386 (36.2) | 215 (46.3) | 359 (62.4) |
| Race, n (%) |  |  |  |  |  |  |  |
| Caucasian | 603 (100.0) | 152 (44.2) | 0 (0.0) | 175 (81) | 791 (81.3) | 465 (100) | 426 (74.1) |
| Asian | 0 (0.0) | 178 (51.8) | 248 (100) | 4 (1.8) | 14 (1.3) | 0 (0.0) | 10 (1.7) |
| African American | 0 (0.0) | 0 (0.0) | 0 (0.0) | 18 (8.3) | 110 (12.3) | 0 (0.0) | 59 (10.2) |
| Hispanic | 0 (0.0) | 0 (0.0) | 0 (0.0) | 15 (6.9) | 147 | 0 (0.0) | 76 (13.2) |
| Multi-racial | 0 (0.0) | 14 (4) | 0 (0.0) | 4 (1.8) | 5 (3.6) | 0 (0.0) | 2 (0.3) |
| Native Americans | 0 (0.0) | 0 (0.0) | 0 (0.0) | 0 (0.0) | 0 (0.0) | 0 (0.0) | 1 (0.15) |
| Pacific Islanders | 0 (0.0) | 0 (0.0) | 0 (0.0) | 0 (0.0) | 0 (0.0) | 0 (0.0) | 0 (0.0) |

**Supplementary Table 2:**

**Supplementary Table 2A: Likelihood scores for symptom dynamic paths.** For each path we illustrate the path likelihood used to identify symptom dynamic paths. Symptom dynamic paths between a baseline and 8 week strata are highlighted in green based on highest likelihood score. For example, the symptom dynamic path between A1 and C1, is A1 🡪 B1 🡪 C1 as the path has likelihood greater than other paths between A1 and C1. The ranges of depression severity scores in each cluster are as follows: A1 [14–18], A2 [19–24], A3 [25–39]; B1 [0–8], B2 [9–15], B3 [16–31]; C1 [0–7], C2 [8–15], and C3 [16–34]. Finally, we do not have a symptom dynamic path from A1 🡪 C3 via any of the 4 week strata as the paths had fewer than 10% of subjects from A1.

**Supplementary Table 3:** For each symptom dynamic path, and prognostic symptom, we illustrate the median score on the paths.

| Symptom Dynamic path | A1->B1->C1 | A1->B2->C2 | A2->B1->C1 | A2->B2->C2 | A2->B3->C3 | A3->B1->C1 | A3->B2->C2 | A3->B3->C3 |
| --- | --- | --- | --- | --- | --- | --- | --- | --- |
| Most likely outcome | Remission | No response | Remission | Response without remission | No response | Remission | Response without remission | No response |
| Depressed Mood | 2->0->0 | 2->1->1 | 3->1->0 | 3->1->1 | 3->2->1 | 3->0->0 | 3->1->1 | 3->3->2 |
| Psychic Anxiety | 2->1->0 | 2->1->1 | 2->1->0 | 2->1->1 | 2->2->1 | 3->1->0 | 3->1->1 | 3->2->2 |
| Guilt Feelings and Delusions | 2->0->0 | 2->1->1 | 2->-0->0 | 2->1->1 | 2->2->1 | 2->0->0 | 2->1->1 | 2->2->2 |
| Work and Activities (Interests) | 2->0->0 | 2->2->1 | 2->-0->0 | 2->1->1 | 3>2->1 | 3->0->0 | 2->1->1 | 3->2->2 |

**SUPPLEMENTARY METHODS**

**Data Sources**

**Training:** Two independent trial datasets were used to develop the prognoses model. The Pharmacogenomics Research Network Antidepressant Medical Pharmacogenomics Study (PGRN-AMPS, NCT 00613470) was a single-arm, open trial designed to assess antidepressant effects of citalopram/escitalopram (selective serotonin reuptake inhibitors (SSRIs)) over 8 weeks in adults (aged 18−84 years) with MDD, and to examine metabolomic and genomic predictors of those outcomes ^1^. Subjects were recruited from primary and specialty care clinics from March 2005 to May 2013. Psychiatric diagnoses were confirmed using modules A, B (screen-only version), and D of the Structured Clinical Interview for DSM-IV (SCID) ^2^. Clinical and demographic variables from the PGRN-AMPS dataset were assessed at baseline using standardized questionnaires. Data from complete cases (baseline, 4-, and 8-week data) of the International SSRI Pharmacogenomics Consortium (ISPC) were used to test the reproducibility of patterns of depressive symptom response inferred in the PGRN-AMPS study ^3^. Descriptions of the ISPC studies have been previously published ^4,5^. Briefly, the ISPC dataset comprised pooled data from 7 clinical trials of SSRIs for depression carried out in North America, Europe, and Asia, in order to examine genetic factors underlying variation in antidepressant responses ^5^. Of the 998 ISPC subjects, we used data from 344 (178 Asians and 152 Caucasians) subjects who were treated with citalopram/escitalopram and had data at 4 and 8 weeks. Only complete cases were considered given our explicit goal of modeling longitudinal symptom responses to study drugs, conditioned on baseline depression severity and changes in depressive symptoms at intermediate time-points. For studying the antidepressant’s effects, we used data from 575 subjects of Eli Lilly’s HMCB (NCT00036335), HMBV (NCT00062673), HMCR (NCT00073411), HMFA (NCT00406848) and HMFS (NCT00536471) studies treated with pill placebo.

**Testing:** For external validation of the prognosis rules derived using patients treated with citalopram/escitalopram, we used three cohorts of patients. First, ISPC patients (N = 248) treated with other SSRI (fluoxetine, paroxetine and sertraline) and Eli Lilly outpatient subjects treated with escitalopram (N = 216). Second, 1067 outpatient subjects of Eli Lilly’s HMCB (NCT00036335), HMBV (NCT00062673), HMCR (NCT00073411), HMFA (NCT00406848) and HMFS (NCT00536471) studies treated with serotonin-norepinephrine reuptake inhibitors (SNRI). Finally, 465 inpatient subjects of the Munich antidepressant response signature study (MARS) treated with combinations with an SSRI plus TCA or an SNRI plus TCA. **Supplementary Table 1** summarizes the social and demographic characteristics of included subjects from each of the three datasets.

**Clinical Outcomes**

Depressive symptoms were measured using the 17-item Hamilton Depression Rating Scale (HDRS ^6^). Remission was defined as a HDRS score ≤ 7 ^6^ at 8 weeks. While remission was defined using scores only at 8 weeks, response was defined as a ≥50% reduction in HDRS total scores from baseline after either 4 or 8 weeks of treatment. Across the three datasets, 60−66% of subjects responded and 40−42% remitted at 8 weeks.

**Analysis Workflow**

A machine learning workflow comprising tasks a – f across 5 stages was developed to predict eventual treatment outcomes using a set of individual depressive items with homogeneity in their longitudinal response.

1. **Background: Patient Clustering and Stratification by Sex:** Unsupervised learning (Gaussian mixture models) was used to generate patient stratification (clusters) by sex at baseline, 4- and 8 weeks based on their total depressive symptom severity – with replication in PGRN-AMPS, STAR*D and ISPC trials ^3,7^. This decision for sex-stratified analyses was based on sex-differences in remission rates in STAR*D ^4^, sex-differences in top biological predictors of antidepressant treatment outcomes in our prior work ^3,7^, and results of a recent systematic review showing sex-dependent effects on trajectories of depressive symptom change ^8^. We did not consider the effects of drug dose/plasma drug levels given that total depression severity in the clusters at all time-points was not associated with either measure in PGRN-AMPS subjects ^7^.

In each of the 3 datasets, there were three clusters of patients based on total depressive symptom scores at baseline (labeled A1, A2, A3), 4 weeks (B1, B2, B3), and 8 weeks (C1, C2, C3). The ranges of depression severity scores for both scales are as follows: For the QIDS-C: A1 [7—12], A2 [13—16], A3 [17—25]; B1 [0—6], B2 [7—11], B3 [12—25]; C1 [0—5], C2 [6—11], C3 [12—24]. For the HDRS: A1 [14—18], A2 [19—24], A3 [25—39]; B1 [0—8], B2 [9—15], B3 [16—31]; C1 [0—7], C2 [8—15], C3 [16—34]. With this purely data-driven approach across all three datasets, all patients in C1 cluster achieved remission, and all patients in C3 cluster were non-responders, i.e., failed achieve remission or response. 87% of patients in C2 cluster achieved response without remission (remaining 13% were non-responders). The clusters (A1,…,C3) inferred at each time-point served as nodes of the probabilistic graph which were then used to study longitudinal effects of antidepressants.

1. **Stage – 1 Model Total Depressive Symptom Dynamics Paths:** Probabilistic graphs (expressed as a hidden Markov model) with forward algorithm were used to explore all possible paths connecting a given baseline cluster to patient clusters at 4 weeks, and then from 4 weeks to 8 weeks. The likelihood of each path was computed using the recursive forward algorithm by defining the graph as a hidden Markov model ^7^. The “most likely” paths for each of the 9 pairs of baseline and 8-week clusters (e.g., A1, C1) were chosen based on their having the highest likelihood scores, provided they had at least 10% of patients of the cluster from which patients originated. These unique “most likely” paths were subsequently referred to as *symptom dynamics paths*.

The forward algorithm is a recursive algorithm to compute the likelihood of a path given a starting state, transition probabilities (from previous hidden state to current hidden state), observation probabilities for a given hidden state, and path probability until reaching the current state. In the forward algorithm formulation ($P_{O}\left( C_{t} \right)= \sum_{t\in T} p\left( O | C_{t} \right)P_{O}(C_{t-1})p(C_{t-1}\to C_{t})$) for computing the path probability at a cluster *C_t_* in time *t* and a given observation *O*, , $p\left( O | C_{t} \right)$ is the probability of observation O in *C_t_,* $P_{O}(C_{t-1})$ is the probability of path until reaching *C_t_* from cluster *C_t-1_* at time t-1, and $p(C_{t-1}\to C_{t})$ is the probability of transition from cluster *C_t-1_ to C_t_*.

A plain language example of this formulation used this work is as follows: Let us suppose that we have a path from A3 (baseline) 🡪 B3 (4 weeks) 🡪 C3 (8 weeks), having observed non-response at B3 and C3. We first compute the path probability of patients traversing from A3 🡪 B3 and observing non-response at B – in this case $p(C_{t-1}\to C_{t})$ is the fraction of patients traversing from A3 🡪 B3 (from baseline (t-1) to 4 weeks(t)), and $p\left( O | C_{t} \right)$ is the probability of non-response at B3 among patients who have traversed from A3 to B3. Then, we want to compute the path probability in reach C3 via B3, having originated from A3. Now, $p(C_{t-1}\to C_{t})$ is the fraction of those who transition from B3 to C3 having originated at A3, $p\left( O | C_{t} \right)$ is the probability of non-response among patients in C3 who have transitioned from A3 🡪 B3, and $P_{O}(C_{t-1})$ is the probability computed from first transition (A3 🡪 B3) and having observed non-response as the outcome at B3. This forward algorithm computation can continue beyond 3 time-points.

1. **Stage – 2 Identifying Core Depressive Symptoms:** To extract homogeneous patterns of antidepressant response, “core depressive symptoms” were defined using three criteria: 1) similar response patterns at all time-points, 2) low inter-individual variability, and 3) patterns of change that were statistically distinct within each of the symptom dynamic paths (inferred in Stage 1 using total depression severity scores). First, unsupervised machine learning (hierarchical clustering with complete linkage) was used to identify individual QIDS-C and HDRS scale items with similar rating patterns (clustered together with a common parent in the tree, except the common parent of the entire hierarchy) within the patient clusters at baseline, 4 weeks, and 8 weeks. Second, we identified symptom clusters wherein clinician ratings for each of the scale items at baseline had a nonzero median and low inter-individual variability. A given item was defined as having low inter-individual variability if the chi-square test for the distribution of clinician ratings was significant after multiple comparisons, with the null hypothesis being that the distributions of ratings for that item were equal. Third, the Kolmogorov-Smirnov test was used to determine if there were statistically significant differences in the distributions of core symptom scores at 4 weeks between each of the symptom dynamic paths leading to non-response, response, and remission at 8 weeks, from a given baseline cluster. The variation in these core symptom’s scores within specific symptom dynamic paths were visualized using average smoothing kernels.
2. **Stage – 3: Assessing Antidepressant Effects on Core Depressive Symptoms:** The Mann-Whitney U-test was used to assess whether the severity of the core depressive symptoms (expressed as a rank order) changed significantly as a likely response to antidepressant treatment between two consecutive time-points on a given symptom dynamics path. The rank order test was constructed as follows. **Rank order test construction:** For example, consider a pair of clusters from consecutive time-points (A1 and B1) on a given path (A1 🡪 B1 🡪 C1), and a specific item from the HDRS (e.g., depressed mood). We identified placebo-pill treated subjects on the same path (i.e., assigning patients to the clusters with same depression severity range as citalopram/escitalopram treated patients). Then we tested if the clinician ratings of severity of the same item were significantly different between patients in the B1 cluster of patients treated with citalopram/escitalopram and those of placebo-pill treated patients. If the p-value (with Bonferroni correction for multiple comparisons due to multiple core symptoms) was significant, then we conclude that the changes in clinician ratings of depressive items’ observed severity were more likely due to antidepressants than to chance.
3. **Stage – 4: Establishing Prognostic Effects of Core Depressive Symptoms:** This step defined the minimum number of core symptoms and levels of improvement in the core symptoms needed at 4 weeks (given a specific baseline cluster) to achieve specific outcomes at 8 weeks. First, the threshold of improvement/failure to improve was chosen based on changes in median scores on symptom dynamic paths between a baseline and 4-week cluster. Second, a chi-square test was conducted on a table comprising the number of core symptoms that exceeded (or failed to exceed) the threshold at 4 weeks, versus the outcome labels (e.g., remitters vs. non-remitters, or responders vs. non-responders). If the chi-square test’s p-value was significant for remission or response/non-response, we computed the probability of the outcome based on how many symptoms had to exceed (or failed to exceed) the threshold. If the p-value was not significant, no conclusions about treatment outcome based on changes in any number of core symptoms were possible. Standard deviations (SD) of the probabilities were established by creating 5 random subsets (maintaining the same proportions of patients who achieved remission/response/non-response), and with 10 repetitions of 5 different random subsets. We then computed the accuracy and odds ratio (OR) of the most-likely outcome expected at 8 weeks in patients who transitioned from a baseline stratum to a stratum at 4 weeks. The OR (and associated p-value) represents the odds that the expected treatment outcome at 8 weeks will occur if patients are covered by the prognoses rule, compared to the odds of the same outcome occurring in patients not covered by the prognoses rule.
4. **Stage – 5: Predict Clinical Outcomes from Early Change in Core Depressive symptoms:** We used the prognoses rules derived from citalopram/escitalopram treated subjects in PGRN-AMPS and ISPC studies to derive the prognoses in patients from independent studies as shown in Table 1. Just as in stage – 4 of the workflow, we report the accuracies and OR (and associated p-value) of the most-likely outcome by comparing the frequency of the outcomes in patients who conformed to the prognoses rules versus those who did not.

Tasks (a) and (c) were *in-situ* (non-time-varying) inferential tasks, whereas task (b) was a longitudinal inferential task requiring conditional dependencies (motivating the use of probabilistic graphs), and task (e) was a predictive task requiring supervised learning methods. Therefore, addressing these sequential tasks required multiple statistical/machine learning methods (Fig. 1), details of which (including the approach of forward algorithm construction in task (b), and cross-validation in training prediction models in task (e)) are explained in Supplementary Section 1.

**REFERENCES**

1. Ji Y, Biernacka JM, Hebbring S, et al. Pharmacogenomics of selective serotonin reuptake inhibitor treatment for major depressive disorder: genome-wide associations and functional genomics. *Pharmacogenomics J.* 2013;13(5):456-463.

2. First MB, Spitzer, Robert L, Gibbon Miriam, and Williams, Janet B.W. Structured Clinical Interview for DSM-IV Axis I Disorders, Clinician Version (SCID-CV). *American Psychiatric Press Inc.* 1996.

3. Athreya AP, Neavin D, Frye MA, et al. Factor Graphs Identify Sex-Specific Antidepressant Response Profiles: Citalopram/Escitalopram As Molecular Probes For Subgroups Of Major Depressive Disorder Patients. *Clinical Pharmacology & Therapeutics.* 2018;In Press.

4. Trivedi MH, Rush AJ, Wisniewski SR, et al. Evaluation of outcomes with citalopram for depression using measurement-based care in STAR*D: implications for clinical practice. *Am J Psychiatry.* 2006;163(1):28-40.

5. Biernacka JM, Sangkuhl K, Jenkins G, et al. The International SSRI Pharmacogenomics Consortium (ISPC): a genome-wide association study of antidepressant treatment response. *Transl Psychiatry.* 2015;5:e553.

6. Hamilton M. A rating scale for depression. *J Neurol Neurosurg Psychiatry.* 1960;23:56-62.

7. Athreya AP, Banerjee SS, Neavin D, et al. Data-Driven Longitudinal Modeling and Prediction of Symptom Dynamics in Major Depressive Disorder: Integrating Factor Graphs and Learning Methods. Paper presented at: IEEE International Conference on Computational Intelligence in Bioinformatics and Computational Biology2017.

8. Musliner KL, Munk-Olsen T, Eaton WW, Zandi PP. Heterogeneity in long-term trajectories of depressive symptoms: Patterns, predictors and outcomes. *J Affect Disord.* 2016;192:199-211.
